# Macrophage-Mediated Antibody Dependent Effector Function in Aggressive B-Cell Lymphoma Treatment is Enhanced by Ibrutinib via Inhibition of JAK2

**DOI:** 10.3390/cancers12082303

**Published:** 2020-08-15

**Authors:** Verena Barbarino, Sinika Henschke, Stuart James Blakemore, Elena Izquierdo, Michael Michalik, Nadine Nickel, Indra Möllenkotte, Daniela Vorholt, Linda Müller, Reinhild Brinker, Oleg Fedorchenko, Nelly Mikhael, Tamina Seeger-Nukpezah, Michael Hallek, Christian P. Pallasch

**Affiliations:** 1 Department I of Internal Medicine, Center for Integrated Oncology (CIO) Köln-Bonn, University of Cologne, Kerpener Str. 62, 50937 Cologne, Germany; Verena.barbarino@uk-koeln.de (V.B.); sinika.henschke@sf.mpg.de (S.H.); stuart.blakemore@uk-koeln.de (S.J.B.); elena.izquierdo-alvarez@uk-koeln.de (E.I.); michael.michalik@uk-koeln.de (M.M.); Dr.Nadine.Nickel@gmail.com (N.N.); indra.moellenkotte@uk-koeln.de (I.M.); danielav@miltenyibiotec.de (D.V.); linda.mueller@uk-koeln.de (L.M.); reinhild.brinker@uk-koeln.de (R.B.); oleg.fedorchenko@uk-koeln.de (O.F.); nelly.mikhael@uk-koeln.de (N.M.); tamina.seeger-nukpezah@uk-koeln.de (T.S.-N.); michael.hallek@uk-koeln.de (M.H.); 2 Cologne Excellence Cluster for Cellular Stress Responses in Ageing-Associated Diseases (CECAD), Center for Molecular Medicine Cologne (CMMC), University of Cologne, Joseph-Stelzmann-Str. 26, 50931 Cologne, Germany

**Keywords:** Ibrutinib, JAK, ruxolitinib, B-cell lymphoma, macrophage, phagocytosis, ADCP, therapeutic antibody

## Abstract

Targeted inhibition of Bruton’s Tyrosine Kinase (BTK) with ibrutinib and other agents has become important treatment options in chronic lymphocytic leukemia, Waldenström’s Macroglobulinemia, Mantle cell lymphoma, and non-GCB DLBCL. Clinical trials combining small molecule inhibitors with monoclonal antibodies have been initiated at rapid pace, with the biological understanding between their synergistic interactions lagging behind. Here, we have evaluated the synergy between BTK inhibitors and monoclonal antibody therapy via macrophage mediated antibody dependent cellular phagocytosis (ADCP). Initially, we observed increased ADCP with ibrutinib, whilst second generation BTK inhibitors failed to synergistically interact with monoclonal antibody treatment. Kinase activity profiling under BTK inhibition identified significant loss of Janus Kinase 2 (JAK2) only under ibrutinib treatment. We validated this potential off-target effect via JAK inhibition in vitro as well as with CRISPR/Cas9 JAK2^−/−^ experiments in vivo, showing increased ADCP and prolonged survival, respectively. This data supports inhibition of the JAK-STAT (Signal Transducers and Activators of Transcription) signaling pathway in B-cell malignancies in combination with monoclonal antibody therapy to increase macrophage-mediated immune responses.

## 1. Introduction

Inhibition of Bruton’s Tyrosine Kinase (BTK) in the treatment of B-cell malignancies has been an exemplary story of functional understanding of disease pathogenesis translating to superior survival rates [1,2,3,4]. Moreover, inhibition of BTK is currently also being evaluated as a therapeutic strategy for patients with severe COVID-19 disease [5,6]. BTK is localized in proximity of the B-cell receptor (BCR), forming a signalosome complex upon BCR activation with other BCR associated kinases (BAK); LYN, SYK, and phosphoinositide-3-kinase (PI3K) [7,8]. The first generation BTK inhibitor (BTKi) ibrutinib covalently binds its target, which leads to prolonged lymphocytosis via reduced malignant B-cell homing capacity and chemokine signaling [9]. However, ibrutinib also has high affinity to other Tec kinases, leading to the development of second generation BTKis, such as tirabrutinib and acalabrutinib, which are highly selective for BTK [10,11]. 

After initial success of ibrutinib in early phase clinical trials as a single agent in the relapse/refractory setting, clinical trials were quickly initiated in combination with other frontline agents, namely monoclonal antibodies targeting CD20 (anti-CD20; such as rituximab) [12,13,14,15]. The most recent phase 3 trial in previously untreated chronic lymphocytic leukemia (CLL) offers superior survival in comparison to the chemo-immunotherapeutic (CIT) regimen fludarabine cyclophosphamide and rituximab (FCR) [12]. Rituximab elicits its therapeutic effect via direct cell killing, complement activation, and Fc-mediated antibody dependent cellular cytotoxicity (ADCC) [16]. In addition, CIT has been previously shown to induce an acute secretory phenotype leading to increased macrophage mediated antibody dependent cellular phagocytosis (ADCP), in a humanized mouse model of B-cell lymphoma [17,18,19]. Therefore, it is important to evaluate the synergistic interaction of BTK inhibition and monoclonal antibody treatment in the context of the tumor microenvironment (TME). 

Pre-clinical studies assessing the synergistic interaction between ibrutinib and monoclonal antibodies have provided conflicting results, with a number of studies reporting that ibrutinib not only has no impact on antibody-mediated effects, rather a negative effect specifically on natural killer (NK)-cell mediated effects of the antibody [20,21,22,23]. Downregulation of CD20 on CLL cells by ibrutinib has additionally been described in co-culture systems and may be interpreted as a potential antagonistic mechanism [24,25]. However, combination therapy of ibrutinib and anti-CD20 antibody has proven to be clinically highly effective especially for unfit patients with CLL [26,27]. As the antibody therapy relies on several independent effector mechanisms antibody-dependent cellular phagocytosis has been previously underestimated [28]. The effector function of macrophages is highly dependent on interaction within the tumor microenvironment. Therefore, we employed a reliable humanized mouse model of “Double Hit-Lymphoma” for functional elucidation of ibrutinib in antibody combination therapy [17,18,19].

Here, we show that ibrutinib synergizes with multiple monoclonal antibodies in vitro and in vivo via increased macrophage mediated ADCP. We screened the kinase activity of malignant B-cells under ibrutinib, tirabrutinib, and acalabrutinib treatment, identifying Janus Kinase 2 (JAK2) as having significantly reduced activity under ibrutinib, whilst second generation BTKis did not similarly inhibit JAK2 activity. Finally, we show using CRISPR/Cas9 knockouts and a kinase inhibitor library that loss of JAK2 as well as inhibition using tofacitinib leads to increased ADCP in vitro, whilst superior survival of JAK2 knockout was observed in combination with monoclonal antibody therapy in vivo. 

## 2. Material and Methods

### 2.1. Cell Lines and Primary Patient Material

The study was approved by the ethical commission of the medical faculty of the University of Cologne (reference no. 13-091) and an informed written consent was obtained from all patients. Primary CLL patient samples were isolated from peripheral blood as previously described [29]. To isolate peritoneal macrophages, 8 to 24 weeks old wild type and BTK^−/−^ C57BL/6 mice (Charles River, Willmington, MA, USA) were injected *i.p.* with thioglycolate and macrophages obtained via peritoneal lavage after four days [18]. BTK^−/−^ C57BL/6 mice were generated by BTK^−/−^ mice backcrossed to C57BL/6J background [30]. Peritoneal macrophages and the murine macrophage cell line J774A.1 were cultured in DMEM (Gibco, Thermo Fischer Scientific, Waltham, MA, USA) supplemented with 10% fetal bovine serum (FBS, Biochrom GmbH, Berlin, Germany) and 1% Pen/Strep (Gibco, Thermo Fischer Scientific). The human- MYC/BCL2 (hMB) cell line (strain 102), generated by Leskov et al., was cultured in B-cell culture medium (BCM) composed of a 1:1 ratio of Iscove’s Modified Dulbecco’s Medium (IMDM) and DMEM, supplemented with 10% FBS, 1% P/S, 1% GlutaMAX and 1% β-Mercaptoethanol [19].

### 2.2. Antibody-Dependent Cellular Phagocytosis (ADCP Assay)

J774A.1 macrophages were cultivated in 96-well plates at 1 × 10^4^ cells per well. After 4 h of incubation at 37 °C, 1.5 × 10^5^ hMB “double-hit” lymphoma or CD19^+^ B-cells derived from CLL patients per well were co-cultured with respective macrophages. Subsequently, this co-culture was treated for 17 h with tyrosine kinase inhibitors and monoclonal antibodies in combination or as mono treatment. To stimulate the ADCP of human cells the human specific anti-CD52 monoclonal antibody alemtuzumab (Genzyme, Cambridge, MA, USA; 10 µg/mL), anti-CD20 obinutuzumab (GA101, Roche, Basel, Switzerland; 1 µg/mL), and anti-CD20 rituximab (Roche, Basel, Switzerland; 20 µg/mL) antibodies were used. Primary human CLL cells were stained with CD19 (fluorescein isothiocyanate (FITC), #363008, BioLegend, San Diego, CA, USA) fluorescent antibody for 15 min at 4 °C before measurement by flow cytometry. Each condition was performed with five replicates. For determination of ADCP GFP^+^, target cells were analyzed using a MACSQuant VYB flow cytometer (Miltenyi Biotec, Berg. Gladbach, Germany). The percentage of ADCP was calculated as follows:

100 − (100 × (cells/µL treated/cells/µL untreated))

### 2.3. Phagocytosis F4-80 Staining

To determine the amount of phagocyted hMB cells by macrophages, J774A.1 were plated out in a density of 1 × 10^5^ cells in 1ml media 4 h prior to addition of target cells. Then 1.5 × 10^6^ hMB cells in 1 mL media, 250 μL alemtuzumab (10 μg/mL), and 250 μL of 0–20 μM ibrutinib (Bertin, M.-le-Bretonneux, France) were added. After 16 h, cells were blocked with 10 µL Fc-receptor blocking reagent. Macrophages were stained with F4-80 allophycocyanin (APC) antibody (BioLegend, San Diego, CA, USA) and cells were analyzed using MACSQuant flow cytometry.

### 2.4. Humanized DHL Mouse Model

For in vivo experiments 8–14 weeks old male NOD.Cg-*Prkdc*^scid^ *II2rg*^tm1Wjl^/SzJ (NSG, Jackson Laboratory, Bar Harbor, Hancock, ME, USA) immunodeficient mice were injected *i.v.* with 1 × 10^6^ hMB cells diluted in 100 µL PBS [19]. Ten days after injection mice were treated *i.p.* on three consecutive days with ibrutinib (30 mg/kg), alemtuzumab (day 1, 1 mg/kg; day 2 and day 3, 5 mg/kg), tirabrutinib (30 mg/kg), or PBS as control. Disease progression was monitored by weekly blood sampling and daily scoring of the mice. Spleen and bone marrow were harvested and dissociated by cell strainers with PBS and lysed with 5 mL ammonium-chloride-potassium (ACK) lysis buffer for 3 min at room temperature (RT). For flow cytometry, samples were stained with F4-80 APC antibody (BioLegend, San Diego, CA, USA). Animal experiments were conducted with permission of the Landesamt für Natur, Umwelt und Verbraucherschutz Northrhine-Westphalia under the file numbers 84-02.04.2016.A119 and Uniklinik Kköln_Anzeige §4.16.009.

### 2.5. Generation of Conditioned Media

For the generation of conditioned media 1.5 × 10^6^ hMB cells/mL were incubated with 0–20 μM of ibrutinib in 10 mL BCM for 24 h. Afterwards the inhibitor was washed off and cells were incubated in 10 mL fresh media for 24 h. Subsequently, the supernatant was taken off for further experiments.

### 2.6. Kinase Activity Profiling by PamChip Microarray

Kinase activity profiles of treated cell lysates were determined via PamChip peptide microarrays on a Pam Station 12 (PamGene International B. V., Netherlands https://www.pamgene.com/en/pamchip.htm). PamChip microarrays contain distinct peptides with 12–15 amino acids representing different proteins. These peptides get phosphorylated depending on the kinase activity in the lysates. Phosphorylated peptides were recognized by phospho-specific PY20 FITC-conjugated antibodies and detected with a charge-coupled device (CCD) camera.

For kinase activity profiling, we used Protein-Tyrosine kinase (PTK) and Serine/Threonine (STK) Chips containing 196 and 140 peptides targeting the main kinase families. For PTK activity profiling 5 µg of total protein concentration was loaded on the chip and dissolved in 4 µL 10× PK buffer, 0.4 µL 100× BSA, 4 mM ATP, 0.6 µL FITC conjugated antibody, 10 mM DTT, 4 µL PTK additive and filled up with distilled water to 40 µL total volume (basic mix). All reagents were supplied by PamGene International B.V. For STK activity profiling, 1 µg of total protein concentration was loaded on the chip and dissolved in 4 µL 10× PK buffer, 0.4 µL 100× BSA, 4 mM ATP, 0.5 µL STK primary Antibody mix and filled up with distilled water to a total volume of 35 µL (basic mix). Moreover, 0.4 µL FITC conjugated antibodies dissolved in 3 µL 10× AB buffer and 26.6 µL water were added after an initial incubation time (detection mix). Prior to sample loading a blocking step was performed loading 30 µL of 2% BSA. To determine the kinetic of kinase activity, the samples were pumped several times (cycles) through the microarray and imaged at certain cycle passages. For all experiments, biological replicates were used. CLL patient samples were measured in technical replicates.

For data analysis, Bio Navigator software (PamGene) was used to calculate the cycle and time dependent signals into a single value for each peptide on the chip (exposure time scaling). Furthermore, outliers due to saturation or insufficient antibody binding were excluded. For analysis, data were log transformed calculating the fold change between treated and untreated, and the *p*-value was calculated using an unpaired Students’ two-tailed t-test. A *p*-value of *p* ≤ 0.05 was accepted as statistically significant. Individual peptides were matched to their representative kinases using a proprietary database (unpublished; PamGene International B. V., Netherlands). In brief, this database ranked the likelihood of peptides belonging to kinases using public databases such as; PhosphoNet, Reactome and UniProt. Volcano plots were produced using the EnhancedVolcano package in RStudio (R version 3.3.1). 

### 2.7. Statistics 

All data was evaluated and graphs generated using GraphPad Prism 8.00 (GraphPad Software, San Diego, CA, USA) and SPSS. Unless otherwise stated, bar graphs represent the mean ± SD of three biological replicates. Box plots show the minimal and maximal value, the 25th and 75th quartiles, and the median. Statistical comparison between groups was performed using the One-way ANOVA multiple comparison test in the ADCP assays, or the Kruskal–Wallis test for non-Gaussian distributed data. Kaplan–Meier survival analysis was performed using pairwise Log-rank (Mantel–Cox) test. Differences were considered statistically significant at *p*-values less than 0.05 (ns = not significant, *p* > 0.05; *, *p* ≤ 0.05; **, *p* ≤ 0.01; ***, *p* ≤ 0.001). 

## 3. Results

### 3.1. Ibrutinib Enhances Macrophage-Mediated Antibody-Dependent Cellular Phagocytosis 

**Figure 1:**
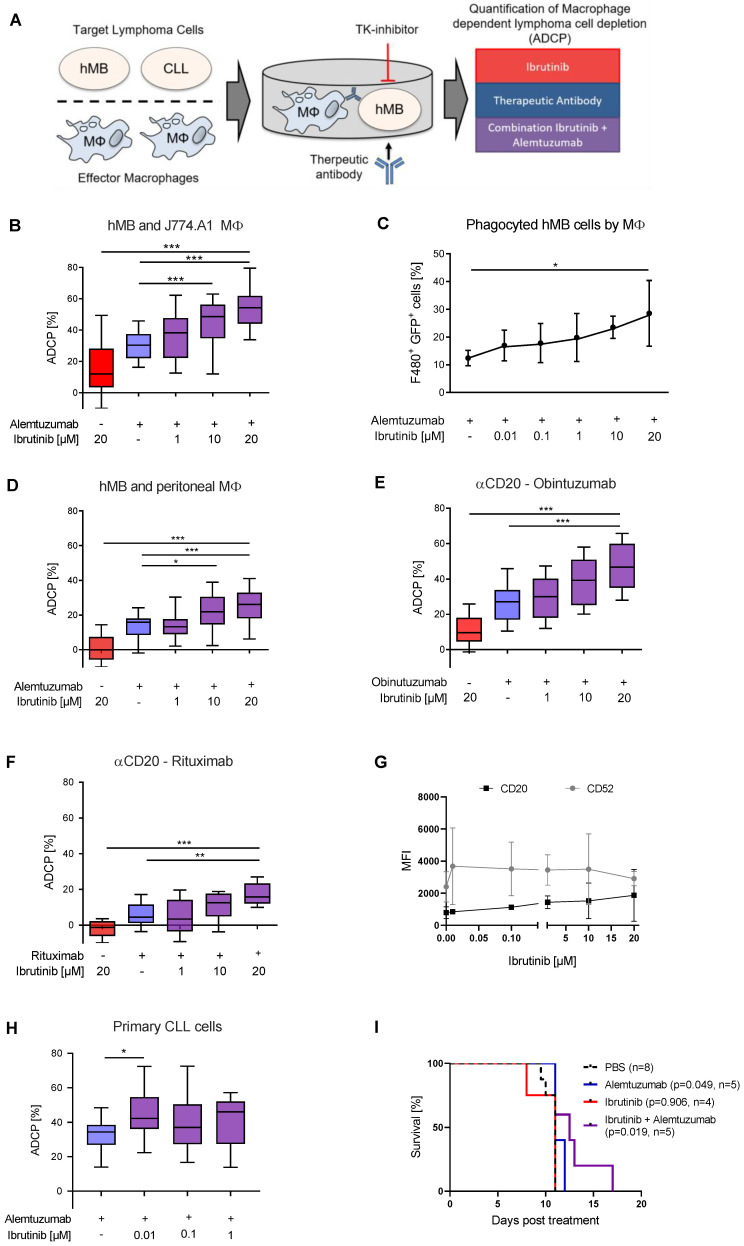
Ibrutinib enhances macrophage-mediated antibody-dependent cellular phagocytosis (ADCP) (**A**) Schematic of Antibody Dependent Cellular Phagocytosis assay. Co-cultures of macrophage effector cells and lymphoma target cells were treated for 17 h with tyrosine kinase (TK) inhibitors and therapeutic antibodies in combination or as monotherapy. Lymphoma target cells were measured by flow cytometry. (**B**) Box plot showing ADCP of hMB “Double-Hit” lymphoma cells and J774A.1 macrophages treated with ibrutinib and alemtuzumab. (**C**) Bar graph showing phagocyted hMB cells by J774A.1 macrophages, measuring GFP^+^ hMB lymphoma cells and F4/80^+^ macrophages in an ADCP model treated with ibrutinib and alemtuzumab. (**D**) Box plot showing ADCP of hMB lymphoma and murine peritoneal macrophages as effector cells treated with ibrutinib and alemtuzumab. (**E**,**F**) Box plot showing ADCP of hMB lymphoma cells and J774A.1 macrophages treated with ibrutinib and anti-CD20 antibody (**E**) obinutuzumab and **(F**) rituximab. (**G**) Expression of CD20 and CD52 surface markers after ibrutinib treatment of hMB lymphoma cells. (**H**) Box plot showing ADCP of CD19^+^ primary chronic lymphocytic leukemia (CLL) patient cells (N = 6) pretreated ex vivo with ibrutinib for 24 h and co-cultured with J774A.1 macrophages. Alemtuzumab treatment was applied for 24 h. The graphic shows the relative macrophage-dependent cell death in the presence or absence of antibody. (**I**) Kaplan-Meier analysis comparing the survival of male hMB transplanted NSG mice receiving ibrutinib and alemtuzumab as mono therapy or in combination. PBS was used as control. The treatment was given *i.p.* 10 days after *i.v.* hMB cell injection. All box plots show the median, the 25th and 75th quartiles, and the minimal and maximal value. All bar graphs display the average and SEM. Unless otherwise stated experiments were performed of at least three biological replicates. (* *p* < 0.05, ** *p* ≤ 0.01 and *** *p* ≤ 0.001).

In order to elucidate the potential synergistic interaction between BTKis and monoclonal antibodies on macrophage-mediated ADCP, we utilized multiple monoclonal antibodies (rituximab, obinutuzumab, alemtuzumab), as well as different types of macrophage effector cells in an ADCP co-culture system ex vivo with the hMB humanized mouse model of “Double-hit” lymphoma as target cells [17,18] (Figure 1A).

Co-treatment of alemtuzumab with serially diluted concentrations of ibrutinib lead to significantly increased antibody-mediated lymphoma cell depletion in a concentration dependent manner (Figure 1B). We did not attribute this significant increase to cell toxicity since at these concentrations the cells retained high cell viability (Figure S1A,B). To verify that ibrutinib specifically enhances phagocytosis of antibody-targeted malignant B-cell lymphoma cells we assessed engulfment of GFP^+^ hMB Double-Hit lymphoma cells into F4/80^+^ J774A.1 macrophages and detected a rising number of F4/80^+^/GFP^+^ cells with increasing ibrutinib concentrations (Figure 1C). Furthermore, this effect was independent of the number of macrophages present under ibrutinib treatment (Figure S1C). Addressing the synergistic effect of ibrutinib and alemtuzumab combination, we performed an ADCP using different concentrations of alemtuzumab mono therapy without significant differences of increasing concentrations but rather an on/off effect by antibody treatment (Figure S1D). We observed similar significant increases in ADCP performing an independent ex vivo experimental setup using primary murine peritoneal macrophages (Figure 1D), whilst also observing concentration dependent antibody-mediated lymphoma cell depletion with the type 1 and 2 anti-CD20 monoclonal antibodies rituximab (Figure 1E) and obinutuzumab (Figure 1F). Thereby, ibrutinib treatment of hMB cells in vitro induced a moderate but non-significant increase of CD20 and CD52 expression (Figure 1G). We furthermore evaluated the effects of ibrutinib on primary leukemic cells from chronic lymphocytic leukemia (CLL) patients. Primary CLL cells pretreated with ibrutinib for 24 h exhibited an increased susceptibility towards alemtuzumab mediated ADCP comparing the ibrutinib treatment with antibody to ibrutinib treatment without antibody therapy (Figure 1H). Importantly, here we observe an increase of ADCP using low ibrutinib concentrations of 0.01 µM. This corresponds to concentrations achieved with oral formulation of 420 mg ibrutinib daily [31].

Finally, we leveraged male NOD.Cg-*Prkdc*^scid^ *II2rg*^tm1Wjl^/SzJ (NSG) mice transplanted with hMB cells and treated them 10 days after transplantation with ibrutinib and alemtuzumab alone and in combination to assess the impact of BTK inhibition on overall survival in vivo. Pairwise Kaplan–Meier analysis between all treatment groups showed a significant increase in overall survival after alemtuzumab treatment as previously shown (alemtuzumab median = 11 days vs. PBS median = 11 days, *p* = 0.049) [8] (Figure 1I). The significant difference observed here is independent of both groups having the same median overall survival, since the log rank test uses observed and expected number of events to calculate the *p*-value, with only the alemtuzumab treated group having mice at risk of death after the median survival point. Moreover, combination treatment of ibrutinib with alemtuzumab significantly increased overall survival of hMB lymphoma carrying mice (ibrutinib + alemtuzumab median = 12.5 days vs. PBS median = 11 days, *p* = 0.019) (Figure 1I). In conclusion, we observed that combinatorial treatment of B-cell lymphoma with ibrutinib and monoclonal antibodies increases macrophage-mediated phagocytosis and improves overall survival in vivo.

### 3.2. Ibrutinib Elicits Increased ADCP Independent of BTK Inhibition

**Figure 2:**
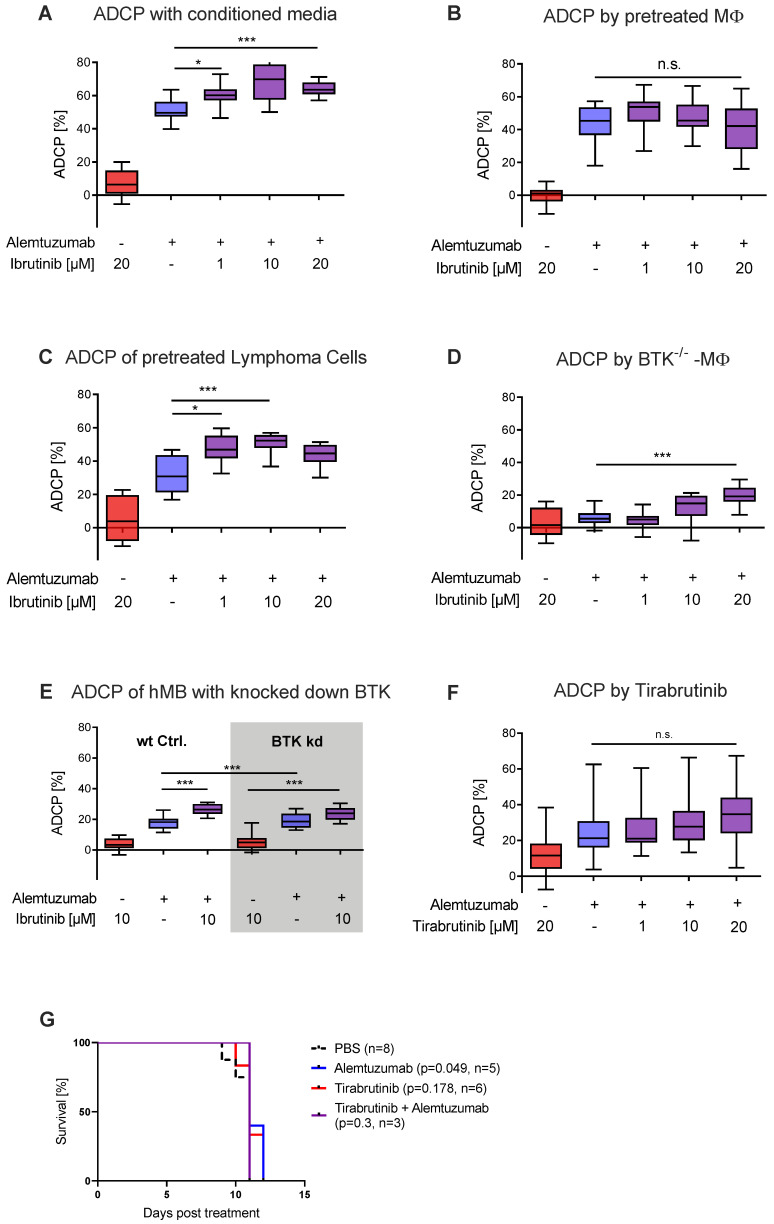
Ibrutinib elicits increased ADCP independent of BTK inhibition (**A**) Box plot showing ADCP of hMB “Double-Hit” lymphoma target cells and J774A.1 macrophages treated with alemtuzumab and conditioned media of ibrutinib pretreated hMB cells. (**B**) Box plot showing ADCP of hMB lymphoma cells co-cultured with ibrutinib-pretreated J774A.1 macrophages, both treated with alemtuzumab. (**C**) Box plot showing ADCP of ibrutinib-pretreated hMB lymphoma cells co-cultured with J774A.1 macrophages, both treated with alemtuzumab (*n* = 2). (**D**) Box plot showing ADCP of hMB lymphoma cells by combination of alemtuzumab with ibrutinib using primary peritoneal macrophages obtained from global BTK^−/−^ mice as effector cells (**E**) Box plot showing ADCP by combination of alemtuzumab with ibrutinib and wild type (wt) hMB lymphoma cells and hMB with knock down in BTK. (**F**) Box plot showing ADCP of hMB lymphoma cells and J774A.1 macrophages treated with ibrutinib and second generation BTK inhibitor tirabrutinib. (**G**) Kaplan–Meier analysis comparing the survival of hMB transplanted male NSG mice receiving tirabrutinib and alemtuzumab as mono therapy or in combination. PBS was used as control. The treatment was given *i.p.* 10 days after *i.v.* hMB cell injection. All box plots show the median, the 25th and 75th quartiles, and the minimal and maximal value. Unless otherwise stated experiments were performed of at least three biological replicates. (* *p* < 0.05 and *** *p* ≤ 0.001).

Previously we have shown that chemotherapy in combination with monoclonal antibody treatment induces an acute secretory activating phenotype (ASAP) leading to increased macrophage-mediated lymphoma cell depletion in vivo [18]. Along these lines, we hypothesized that ibrutinib could be eliciting a similar effect. To analyze the impact of soluble factors released by malignant B-cells, conditioned media from ibrutinib pretreated hMB cells was generated and applied to ADCP co-culture of treatment naïve effector and target cells. Here, we observed a significant increase of ADCP induced by conditioned media obtained from ibrutinib pretreated hMB cells (Figure 2A). To validate whether ibrutinib was eliciting its action via the effector or target cells, we conducted pretreatment in both J774A.1 and hMB cells, followed by co-culture with treatment naïve cells in the ADCP assay. When we pretreated macrophages with ibrutinib and subsequently added treatment naïve hMB target cells to the ADCP assay, we did not detect any significant change in phagocytosis (Figure 2B). In contrast, when we applied ibrutinib pretreated hMB cells to treatment naïve macrophages in co-culture, we observed a significant increase in ADCP (Figure 2C). This suggests that ibrutinib is eliciting its synergistic interaction with monoclonal antibody mainly in the malignant B-cells.

In comparison to second generation BTKis, ibrutinib is not as highly selective for BTK binding, having affinity for other Tec kinases, such as EGFR and JAK2 [11]. Therefore, we interrogated whether ibrutinib mediated its effect through covalently binding its main target BTK, leveraging primary peritoneal macrophages from BTK^−/−^ mice (Figure 2D) and knocking down BTK in hMB cells (Figure 2E, Figure S2A). In these experiments, we observed significant increases in ADCP by ibrutinib for BTK^−/−^ peritoneal macrophages and no significant difference in ibrutinib’s ADCP-enhancing effect between wt hMB cells and hMB cells with a knock down in BTK treated with alemtuzumab, suggesting that one of ibrutinib’s off-target kinases was responsible for the observed effect. To further confirm that BTK was not responsible for this effect, we conducted ADCP assays in vitro using the second generation BTKis tirabrutinib (Figure 2F) and acalabrutinib (Figure S2B) both showing similar levels of phagocytosis and no toxicity (Figure S2C,D). Moreover, combination therapy with alemtuzumab and tirabrutinib in vivo (Figure 2G) did not increase overall survival and did not reduce hMB cells or macrophages in spleen and bone marrow. (Figure S2E–H). In conclusion, we have identified that the ADCP-enhancing effects of ibrutinib were mediated by targeting the malignant B-cell compartment, which induces a secretory component in the malignant B-cells leading to macrophage activation. Furthermore, we have shown that the observed effect is independent of BTK, therefore we hypothesized that the increased ADCP induced by ibrutinib to be associated with its off-target kinases. 

### 3.3. Kinase Activity Profiling Identifies Janus Kinase 2 & 3 as the Main Off-Targets for Ibrutinib vs. Second Generation BTKis

**Figure 3:**
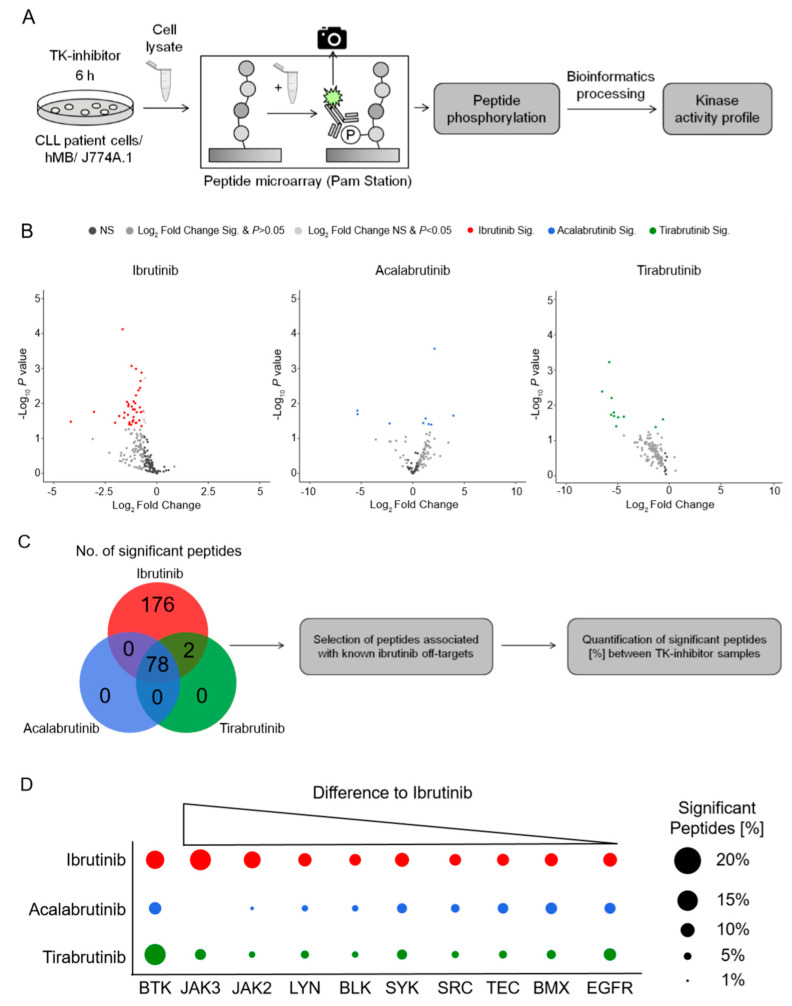
Kinase activity profiling identifies Janus Kinase 2 & 3 as the main off-targets for ibrutinib vs. second generation BTKis (**A**) Schematic of the PamStation approach assessing lysates of treated cells to peptide microarray chip. Peptides on the chip gets phosphorylated depending on the kinase activity of respective lysates. The phosphorylation is detected via fluorescently labelled antibodies. Bioinformatics processing using different databases transform the peptide phosphorylation level into a kinase activity profile. (**B**) Volcano plots of significantly changed peptide phosphorylation after ibrutinib (red, *n* = 6), acalabrutinib (blue, *n* = 3) and tirabrutinib (green, *n* = 3) treatment of hMB lymphoma cells. Each dot represents a kinase peptide substrate represented on the peptide microarray chip. Colored dots indicate significantly altered peptides (two-sided student t-test, *p* ≤ 0.05; log2 fold change ≤ or ≥ 0.5). A negative log2 fold change stands for a downregulation of peptides and a positive log2 fold change for an upregulation compared to the untreated control. Acalabrutinib and tirabrutinib treated lysates were only applied to protein tyrosine kinase (PTK) chips. (**C**) VENN diagram showing all significantly changed peptides of the PTK chips for respective treatments. Based on these significantly changed peptides a kinase activity profile is calculated via bioinformatics processing. (**D**) Graphic showing ibrutinib (red) off-target kinases and its number of significantly changed peptides. Kinase peptide numbers are sorted from the highest difference to acalabrutinib (blue) and tirabrutinib (green) to the lowest. Graphics show biological replicates (*n* = 3).

To identify the off-target effects of ibrutinib that might be responsible for the synergistic interaction observed in Figure 1; Figure 2, we analyzed the kinase activity profiles of hMB cells as well as CLL patient cells under in vitro BTKi treatment after 6 h. Therefore, we used PamChip microarrays, an assay which measures the phosphorylation of protein tyrosine as well as serine/threonine kinases [32] (Figure 3A). Treating hMB lymphoma cells with ibrutinib or tirabrutinib predominantly reduced peptide phosphorylation (Figure 3B; left and right hand panels), whilst acalabrutinib-treated cells displayed both up and downregulated peptides (Figure 3B; central panel). We observed 78 significantly altered peptides, which were shared across all three BTKis, whereas ibrutinib was the only BTKi to downregulate peptides not observed under the treatment of acalabrutinib or tirabrutinib, indicating the lower specificity of ibrutinib compared to second generation BTKis (Figure 3C). To delve deeper into off targets of ibrutinib, we conducted a stratified analysis based on kinases that include the same cysteine residue as BTK [33] (Figure 3D). Importantly, all three BTKis targeted BTK in a similar fashion. Furthermore, we observed an enrichment of significant peptides for Janus Kinase (JAK) 2 and 3, as well as for LYN and BLK under ibrutinib treatment, whilst the other Tec kinases showed minimal changes between BTKi treatments. Likewise, ibrutinib treatment of CLL patient cells enrolled significant downregulation of peptides associated with BTK as well as off-target kinases JAK2/3, LYN, BLK, and other Tec kinases (Figure 3A,B). In conclusion, this data suggests that JAK2 and JAK3 could be the off-target kinases of ibrutinib, and therefore potentially the responsible kinases for the increased macrophage-mediated phagocytic capacity.

### 3.4. JAK2 Inhibition with Ruxolitinib and Tofacitinib Enhances Macrophage Mediated ADCP

**Figure 4:**
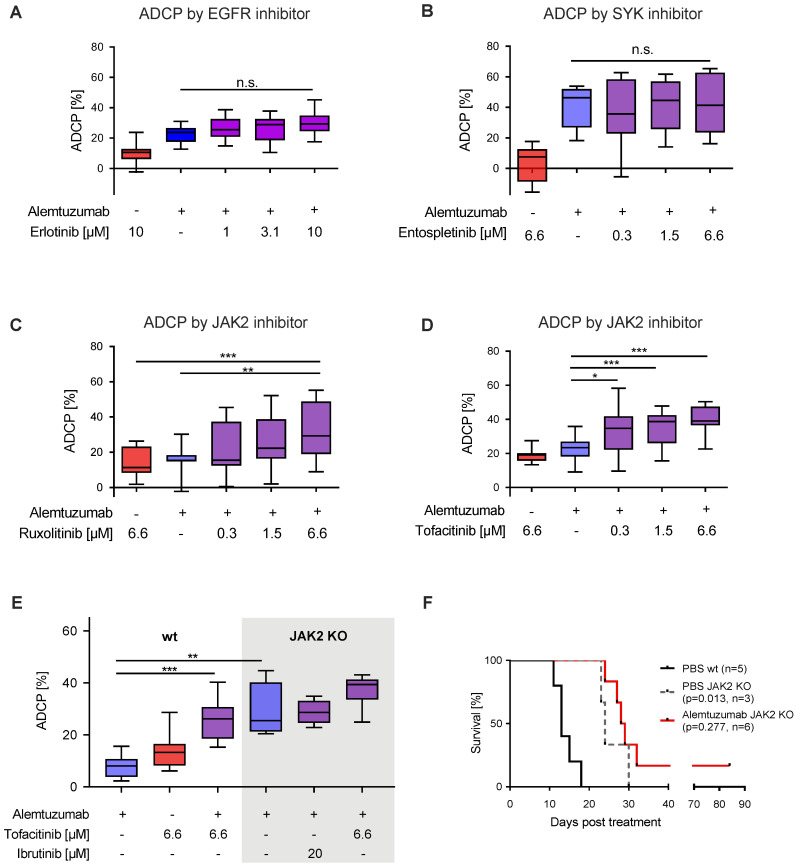
JAK2 inhibition with ruxolitinib and tofacitinib enhances macrophage-mediated ADCP (**A–D**) Box plot showing ADCP of hMB “Double-Hit” lymphoma cells and J774A.1 macrophages treated with alemtuzumab and (**A**) erlotinib (EGFR inhibitor), (**B**) entospletinib (SYK inhibitor, *n* = 2), (**C**) ruxolitinib (JAK inhibitor) and (**D**) tofacitinib (JAK inhibitor). (**E**) Box plot showing ADCP of JAK2^−/−^ vs empty vector control transduced hMB lymphoma target cells and J774A.1 macrophages treated or not treated with JAK2-inhibitor tofacitinib, ibrutinib and alemtuzumab (*n* = 2). (**F**) Kaplan–Meier analysis comparing the survival of wild type (wt) and male JAK2^−/−^ hMB transplanted NSG mice receiving PBS or alemtuzumab. The treatment was given *i.p.* 10 days after *i.v.* hMB cell injection. All box plots show the median, the 25th and 75th quartiles, and the minimal and maximal value. Unless otherwise stated experiments were performed of at least three biological replicates. (* *p* < 0.05, ** *p* ≤ 0.01 and *** *p* ≤ 0.001).

To translate our kinase activity findings back to macrophage mediated ADCP, we generated a modestly sized Tec kinase inhibitor library, conducting concentration dependent ADCP assays in vitro. As expected, inhibition of EGFR (erlotinib; Figure 4A), SYK (entospletinib; Figure 4B), and BMX (CHMFL-BMX-078; Figure S4A), lead to no significant increases in phagocytosis. Importantly, inhibition of JAK1/2 via ruxolitinib (Figure 4C), JAK2/3 via tofacitinib (Figure 4D) and pan-JAK inhibition via SP600125 (Figure S4B) all induced significant increases in macrophage mediated ADCP in a concentration dependent manner. Furthermore, pretreatment of either macrophages or lymphoma hMB cells with ruxolitinib significantly increased ADCP (Figure S4C,D). Notably, all inhibitors did not display direct cytotoxicity or apoptosis induction to lymphoma target cells in the concentrations used (Figure S4E–J). 

To specifically address the functional implications of JAK2 signaling disruption on lymphoma cell susceptibility to ADCP we generated JAK2 deficient lymphoma target cells (Figure S4K). In our ADCP assay JAK2^−/−^ cells showed significantly increased phagocytosis under monoclonal antibody treatment in comparison to JAK2 wild type cells, which could not be improved by co-treatment with either ibrutinib or tofacitinib (Figure 4E). To validate our findings in vivo, we leveraged once again hMB humanized mouse model, comparing wild type hMB cells with JAK2^−/−^ hMB cells with and without alemtuzumab. In this analysis, we observed a clear superior survival of JAK2^−/−^ vs. PBS wild type cells (PBS JAK2^−/−^ median = 24 days vs. PBS wild type median = 13 days, *p* = 0.013) (Figure 4F). Furthermore, alemtuzumab treatment of JAK2^−/−^ cells induced a moderate but not significant increase in survival (alemtuzumab JAK2^−/−^ median = 28.5 days vs. PBS JAK2^−/−^ median = 24 days, *p* = 0.277). In conclusion, we have shown that inhibition of JAK2 facilitates an increased macrophage-mediated lymphoma cell depletion in vitro, with superior overall survival of hMB JAK2^−/−^ transplanted NSG mice in vivo.

## 4. Discussion

We have identified a synergistic interaction between ibrutinib and monoclonal antibody treatment on macrophage mediated ADCP in the treatment of B-cell lymphoma. Interestingly, we observed that this synergy was not mediated by inhibition of BTK as the primary target of ibrutinib but rather was mediated by targeting of the off-target kinase JAK2. Using JAK2 inhibitors instead of ibrutinib, we could recapitulate the synergistic interaction, therefore identifying inhibition of the JAK-STAT signaling pathway as a potential target for antibody combinatorial approaches in the treatment of B-cell malignancies.

While monoclonal antibodies exert various mechanisms of targeting malignant cells in B-cell lymphoma, macrophage ADCP has been identified as a pre-dominant mechanism [18,28]. In this light, previous reports examining ibrutinib in combination with rituximab that did not reveal synergy were mostly focused on NK-cell ADCC or relied on monocyte derived macrophages [20,21,22,23,34]. Exploring effector cell mechanisms is technically challenging since co-culture systems demand high levels of standardization. However, regarding NK-cell dependent ADCC only modest effects of ibrutinib have been observed [20] or even debated to be inhibitory [21].

Here we employed three independent therapeutic antibodies to different targets and respective expression levels and Fc-receptor affinities, two independent macrophage effector cell in vitro models including primary peritoneal macrophages. Moreover, our in vitro ADCP findings were underlined by significantly improved survival in vivo by combination treatment of a humanized mouse model of “Double-Hit” lymphoma [19]. As the used NSG mice are immunodeficient and do not display mature T-cells, B-cells, or NK cells, the main effector cells are macrophages which mediate the antitumor effect of alemtuzumab and ibrutinib [35].

Off-target effects of ibrutinib have been identified recently by independent methodology [33], likewise using Kinobeads the inhibition of TEC kinase family members and particularly BLK by ibrutinib could be shown [36]. However, it remains to be clarified which potential additional kinases beyond BTK are relevant for mediating anti-leukemic effects. 

As for the interpretation of clinical trial data it remains to be clarified if ibrutinib combinations with anti-CD20 antibodies rituximab or obinutuzumab are superior to monotherapy in B-cell lymphoma [37]. Nevertheless ibrutinib/ rituximab combination therapy has superior outcome to FC-R as the previous first-line standard therapy of CLL [12]. Thereby, the beneficial effect of this combination treatment might be independent on altered CD20 expression due to ibrutinib treatment (Figure 1G).

Using second generation BTKis we did not observe a synergistic effect, although the combination of acalabrutinib and obinutuzumab was shown to improve progression-free survival for patients with treatment-naive symptomatic CLL [38]. Here, the high single-agent activity of each drug or ADCP-independent mechanisms of synergy are probably reflecting the superior clinical response in CLL. In our work, we primarily address aggressive Double-Hit lymphoma cells with a potentially less prominent dependence of the malignant B-cell towards sustained BTK signaling. 

As ibrutinib has shown clinical activity in MCL and non-GCB DLBCL, ibrutinib in combination with R-CHOP regimens for treatment of first-line DLBCL revealed a clinical benefit only in younger patients [39]. In Waldenström´s Macroglobulinemia (WM) the combination of rituximab and ibrutinib displayed superior progression-free survival and has been FDA-approved [15]. In WM a constitutively activated JAK-STAT signaling pathway has been linked to secretion and disease progression [40]. As another B-cell receptor signaling related pathway the impact of JAK2-signaling has also been proposed to be relevant in CLL. Here it could be shown that anti-IgM mediated BCR stimulation induces STAT3 activation signaling in CLL [41]. In a clinical trial inhibition of JAK2 by ruxolitinib decreases symptoms [42], however therapeutic impact regarding relevant endpoints such as progression free survival (PFS) and overall survival (OS) remains to be clarified. Finally, Kondo et al. could show that ibrutinib treatment of CLL cells also inhibits STAT3 phosphorylation and described suppression of interleukin-10 (IL10) and programmed cell death ligand 1 (PD-L1) in ibrutinib treated CLL patient cells [43]. In this line a single cell immune profiling of the temporal dynamics of ibrutinib treatment in CLL patient revealed profound modulation of the non-malignant immune cells and particularly up-regulation of inflammatory pathways in myeloid cells [44]. In this context the potential benefits of early phase trials using BTK inhibitors or ruxolitinib in severe COVID-19 patients can be hypothesized to be related to overlapping target specificity and regulation of inflammatory pathways [5,6,45].

In the perspective of B-cell lymphoma we propose JAK2 to be inhibited by ibrutinib and regulating the subsequent phosphorylation of STAT3 and 6 leading to decreased transcription of M2 macrophage phenotype genes [46]. Thereby, we hypothesize JAK2 to interact with the tumor microenvironment leading to re-activation of macrophage effector function. Furthermore, we argue that inhibition of JAK-STAT signaling might sensitize B-cell lymphoma cells towards macrophage mediated ADCP and JAK2 inhibitors should be evaluated and utilized in future treatment concepts of B-cell malignancies. 

## 5. Conclusions

Combination of ibrutinib and monoclonal antibody treatment in the context of B-cell lymphoma provides synergy independent of BTK-inhibition via targeting of JAK2. JAK2 inhibition per se increased therapeutic targeting by ADCP, revealing JAK-STAT signaling as a potential target for development of antibody-containing combinatorial approaches in B-cell malignancies.

## Supplementary Materials

The following are available online at https://www.mdpi.com/2072-6694/12/8/2303/s1.
