# Supplementary material for: Macrophage-Mediated Antibody Dependent Effector Function in Aggressive B-Cell Lymphoma Treatment is Enhanced by Ibrutinib via Inhibition of JAK2
Source: Cancers (Basel). 2020 Aug 15;12(8):2303. doi: 10.3390/cancers12082303 (PMC7465917; doi:10.3390/cancers12082303)
Supplement: Supplementary file 1 [file cancers-12-02303-s001.pdf]

Supplementary material

Macrophage-Mediated Antibody Dependent Effector Function in Aggressive B-Cell Lymphoma Treatment is Enhanced by Ibrutinib via Inhibition of JAK2

Verena Barbarino, Sinika Henschke, Stuart James Blakemore, Elena Izquierdo, Michael Michalik, Nadine Nickel, Indra Möllenkotte, Daniela Vorholt, Linda Müller, Reinhild Brinker, Oleg Fedorchenko, Nelly Mikhael, Tamina Seeger-Nukpezah, Michael Hallek and Christian P. Pallasch

Supplemental Figures

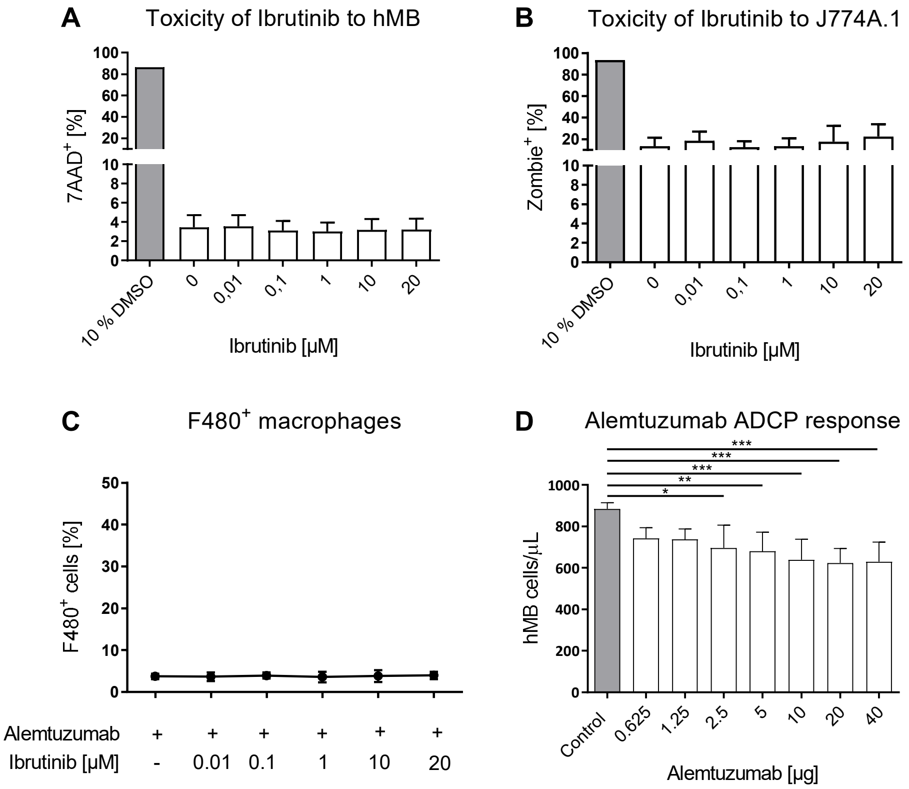

Figure S1. Ibrutinib enhances macrophage-mediated antibody-dependent cellular phagocytosis (ADCP) (**A**) Toxicity staining of ibrutinib treated hMB "Double-Hit" lymphoma cells with 7AAD. (**B**) Toxicity staining of ibrutinib treated J774A.1 macrophages with Zombie staining. (**C**) Bar graph showing F4/80+ J774A.1 macrophages treated with alemtuzumab and different concentrations of ibrutinib. (**D**) Alemtuzumab ADCP response related to antibody concentration. All bar graphs display the average and SEM. (*$p < 0.05$, **$p \leq 0.01$ and ***$p \leq 0.001$).

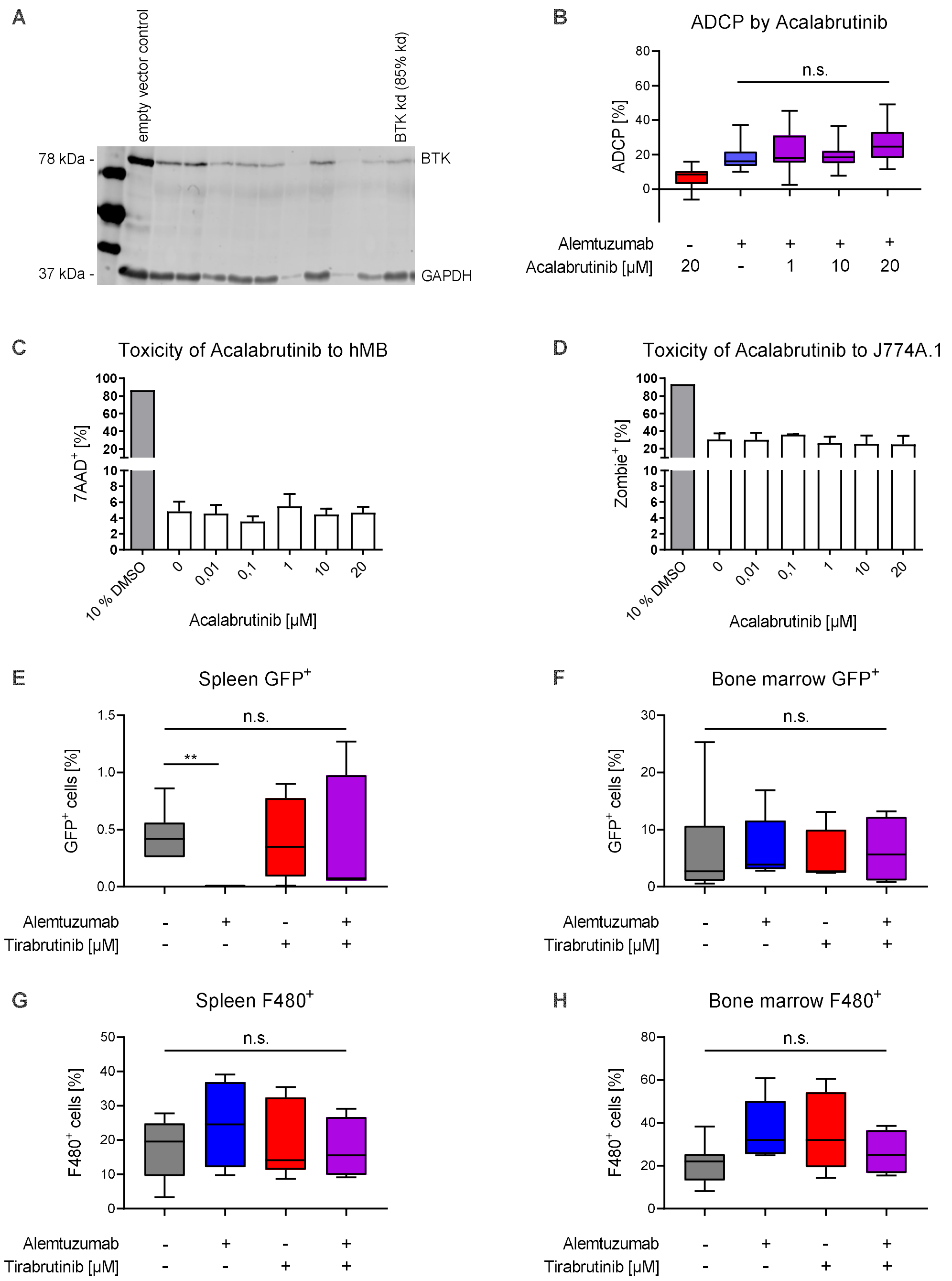

Figure S2. Ibrutinib elicits increased ADCP independent of BTK inhibition (**A**) Western blot analysis of BTK expression in mCHERRY$^+$-sorted control vector-infected versus BTK vector-infected cells. GAPDH serves as loading control. hMB "Double-Hit" lymphoma cells showed a knock down of 85%. (**B**) Box plot showing ADCP of hMB lymphoma cells and J774A.1 macrophages treated with alemtuzumab and acalabrutinib (2nd generation BTKi). (**C**) Toxicity staining of acalabrutinib treated hMB lymphoma cells with 7AAD. (**D**) Toxicity staining of acalabrutinib treated J774A.1 macrophages with Zombie staining. (**E–F**) Box plot showing GFP$^+$ hMB lymphoma cells in (**E**) spleen and (**F**) bone marrow after survival of male hMB transplanted NSG mice treated with alemtuzumab and tirabrutinib in combination or as monotherapy. The treatment was given *i.p.* 10 days after *i.v.* hMB cell injection. (**G–H**) Box plot showing F4/80$^+$ macrophages in (**G**) spleen and (**H**) bone marrow of male hMB transplanted NSG mice with respective treatments. All box plots show the median, the 25th and 75th quartiles and the minimal and maximal value. All bar graphs display the average and SEM. Unless otherwise stated experiments were performed of at least three biological replicates. (**$p \leq 0.01$).

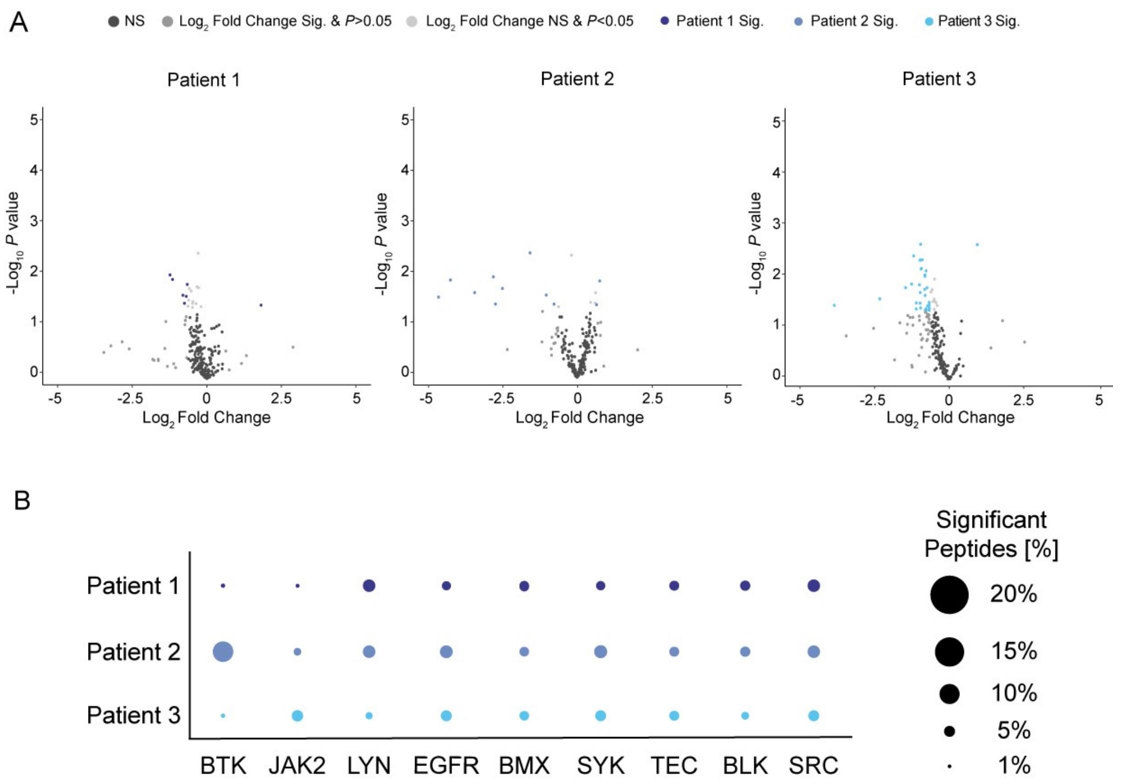

Figure S3. Kinase activity profiling of CLL patient cells identifying the main off-targets for ibrutinib (**A**) Volcano plot of significantly changed peptide phosphorylation after ibrutinib treatment of CLL patient cells. Each dot represents a kinase peptide substrate on the peptide microarray chip. Colored dots indicate significantly altered peptides (two-sided students t-test, $p \leq 0.05$; $\log_2$ fold change ≤ or ≥ 0.5). A negative $\log_2$ fold change stands for a downregulation of peptides and a positive $\log_2$ fold change for an upregulation compared to the untreated control. (**B**) Graphic showing ibrutinib off-target kinases and its number of significantly changed peptides. Graphics show technical replicates ($n = 3$).

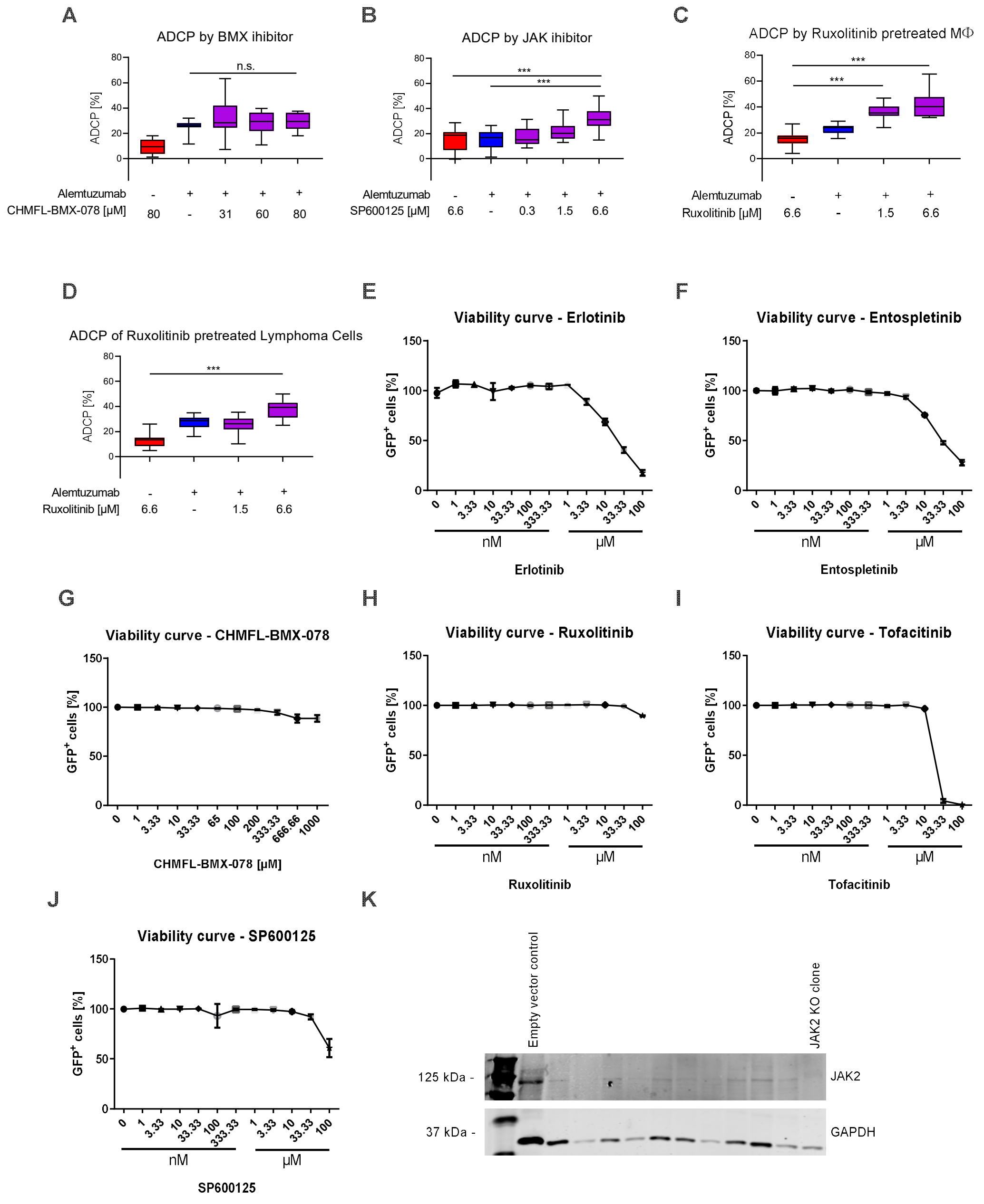

Figure S4. JAK2 inhibition with ruxolitinib and tofacitinib enhances macrophage-mediated ADCP (**A**–**B**) Box plot showing ADCP of hMB "Double-Hit" lymphoma cells and J774A.1 macrophages treated with alemtuzumab and (**A**) CHMFL-BMX-078 (BMX inhibitor, n = 2) or (**B**) SP600125 (JAK inhibitor). (**C**) Box plot showing ADCP of hMB lymphoma cells co-cultured with ruxolitinib-pretreated J774A.1 macrophages, both treated with alemtuzumab. (**D**) Box plot showing ADCP of ruxolitinib-pretreated hMB lymphoma cells co-cultured with J774A.1 macrophages, both treated with alemtuzumab. (**E**–**J**) Viability curve of GFP^+ hMB lymphoma cells treated with (**E**) erlotinib (EGFR inhibitor, n = 2), (**F**) entospletinib (SYK inhibitor), (**G**) CHMFL-BMX-078 (BMX inhibitor), (**H**) ruxolitinib (JAK inhibitor), **(I)** tofacitinib (JAK inhibitor) and (**J**) SP600125 (JAK inhibitor). (**K**) Western blot analysis of JAK expression in $mCHERRY^+$-sorted control vector-infected versus $JAK2^{-/-}$ infected cells. GAPDH serves as loading control. All box plots show the median, the 25th and 75th quartiles and the minimal and maximal value. Viability curves show the mean and SEM. Unless otherwise stated experiments were performed of at least three biological replicates. (***$p \leq 0.001$).

Supplemental Methods

Cell Lysate Preparation and Protein Quantification

For lysate preparation 5 × 10^6 hMB, J774A.1 macrophages and CLL patient cells were treated for 6 h with either 1 µM ibrutinib (Bertin, M.-le-Bretonneux, France), acalabrutinib (Selleckchem, München, Germany) or tirabrutinib (GS4059, Gilead, Foster City, CA, USA). Afterwards cells were washed with PBS and centrifuged for 5 min at 300× g. Next 100 µL M-PER lysis buffer (Mammalian Extraction Buffer, ThermoFisher Scientifc #78503, #78420, #87785 Waltham, MA , USA) with 1 µL Halt Phosphatase Inhibitor Cocktail (100×, Thermo Fischer Scientific #78428) and 1 µL Halt Protease Inhibitor Cocktail, EDTA free (100×, Thermo Fischer Scientific #78437) was given to the cell pellet and incubated for 15 min on ice. Then the cell pellets were centrifuged for 15 min at 16,000 g at 4 °C. The supernatant containing the proteins was transferred into a fresh tube and frozen at −80 °C in 10 µL aliquots. Protein concentration was determined using Pierce BCA Protein Assay (Thermo Fisher, #23225) and measured with microplate reader FluoStar Optima (BMG Labtech, Ortenberg, Germany).

Toxicity Staining

To analyze toxicity, 1 × 10^6 cells in 2 mL medium were incubated with respective tyrosine kinase inhibitor for 24 h. After 24 h, macrophages were stained with Zombie NIR^{M}Fixable Viability Staining diluted 1:100 in PBS and incubated for 15 min in the dark at room temperature (RT). hMB and CLL patient cells were stained with 7AAD diluted 1:100 in PBS for 15 min at 4 °C. 7-AAD toxicity staining was measured by MACSQuant flow cytometer (Miltenyi Biotec, Berg. Gladbach, Germany).

Generation of Crispr Mediated Knock-out in hMB Cells

All guide RNAs for BTK knock out (KO) (#7707 1-4), JAK2 KO (#7572 6-7) and non-target plasmids (#80248) were gifted from John Doench and David Root[1]. First, hMB lymphoma cells were transduced with a virus containing CRISPR mCherry Cas9 guide RNA gifted from Agata Smogorzewska (#99154) and afterwards with the respective KO or non-target guide RNA. The corresponding plasmid was packaged into lentiviral particles using amphotrophic HEK 293T phoenix cells co-transfected with psPAX2 and pMD2.G. Six hours after transfection, the medium was changed and subsequently collected at 24 h intervals till 72 h. Then, the hMB cell line was transduced with respective lentiviral particles by spin infection (800 g, 32 °C, 2 h). After infection for 48 h, cells were cultured and antibiotic selection with 10 µg/mL puromycin was started for at least 2 weeks. After a week single cell sorting and western blot analysis was performed.

Western Blot Analysis

Whole cell pellets were lysed with RIPA buffer (Cell Signalling Technology Cat. Danvers, MA, USA) containing phosphatase Inhibitor cocktail (100×, Thermo Fischer Scientific) and protease inhibitor cocktail, EDTA free (100×, Thermo Fischer Scientific). 10 µg of total protein of each sample was separated on 10% SDS-PAGE, according to antibody manufacturer's instructions, and transferred onto a nitrocellulose membrane (GE Healthcare, Freiburg, Germany). The membranes were blocked 1 h at RT (according to manufactures' protocol) and incubated with the corresponding primary antibody for BTK (D3H5 rabbit mAB #8547, Cell Signaling) and JAK2 (D2E12 XP® rabbit mAB #3230, Cell Signaling) overnight at 4 °C. After washing, membranes were stained with secondary fluorescent dye-labeled antibodies (LI-COR Biotech., Bad Homburg, Germany) for 1 h at RT. Protein bands were detected at 700 or 800 nm using the LI-COR Odyssey infrared imaging system. Protein loading was normalized against GAPDH. Densitometry was performed with Image Studio Lite Ver 5.2 software.
